# Editorial: New insights into the disorder of brain connectivity in schizophrenia

**DOI:** 10.3389/fnimg.2023.1266695

**Published:** 2023-08-29

**Authors:** Weikai Li, Md. Mamun Al-Amin, Aarti Nair

**Affiliations:** ^1^College of Mathematics and Statistics, Chongqing Jiaotong University, Chongqing, China; ^2^MIIT Key Laboratory of Pattern Analysis and Machine Intelligence, Nanjing, China; ^3^Stark Neurosciences Research, Indiana University, Bloomington, IN, United States; ^4^School of Behavioral Health, Loma Linda University, Loma Linda, CA, United States

**Keywords:** schizophrenia, brain connectivity, neuroimaging, machine learning, functional connectivity

## Introduction

The field of mental health has witnessed remarkable advancements as we enter the third decade of the twenty first Century. To highlight the latest progress in mental health research, we have organized a Research Topic on new insights into the disorder of brain connectivity in schizophrenia. The collected articles on this topic focus on neuroscience research that can offer insights into solutions for fostering a culture of awareness, understanding, and solidarity. In particular, there is still more work to be done in elucidating the precise mechanisms underlying schizophrenia research using neuroimaging techniques, especially in the view of the connectome. This Research Topic invites brief contributions from researchers worldwide that describe the current state-of-the-art and outline recent developments and major accomplishments achieved in advancing this field further. The authors were encouraged to identify key challenges within their sub-disciplines and propose strategies for addressing them effectively. This special edition of Research Topic aims to shed light on both the progress made over the past decade in clinical neuroimaging and schizophrenia as well as future challenges while providing a comprehensive overview of the state-of-the-art in clinical neuroimaging. It includes four papers that extensively analyze schizophrenia based on machine learning algorithms and brain connectivity.

## Investigating schizophrenia using machine learning techniques

The study by Keyvanfard et al. focuses on analyzing the brain network using machine learning techniques. They apply independent component analysis (ICA) to the subject's dimension of the functional connectivity (FC) matrices. The study includes 27 individuals with schizophrenia (SZ) and 27 carefully matched healthy controls (HC). The authors use a graph theory approach to evaluate the properties of the functional network. Patients with schizophrenia are found to differ significantly from healthy controls in the visual, ventral attention, and somatomotor subnetworks. Surprisingly, when the same parameters (such as path length, network strength, global/local efficiency, and clustering coefficient) are calculated for the entire brain network using the same data, no significant difference is found. Excitingly, the researchers propose a simple scoring classifier based on two of these subnetworks. This classifier showed acceptable sensitivity and specificity, with an area under the receiver operating characteristic curve of 77.5%. For a deep-learning-based method, Mao et al. propose an unsupervised learning-based generative adversarial network with adaptive normalization for synthesizing T2-weighted MR images from rapidly scanned diffusion-weighted images (DWI). This method provides an effective framework for the downstream schizophrenia diagnostic task or network analysis.

## Brain connectivity-based analysis of schizophrenia

In view of brain connectivity, Xue et al. focus on detecting the aberrant patterns of functional connectivity within and between large-scale cortico-hippocampal networks in first-episode schizophrenia patients. The authors use resting-state functional magnetic resonance imaging (rs-fMRI) data to compare patients with schizophrenia with the healthy control. This study measures the correlation between functional connectivity and score from cognitive tests. Xue et al. reveal that the schizophrenia patients manifest widespread within-network FC alterations of the cortico-hippocampal network, with the decrease of FC involving the precuneus (PREC), amygdala (AMYG), parahippocampal cortex (PHC), orbitofrontal cortex (OFC), perirhinal cortex (PRC), retrosplenial cortex (RSC), posterior cingulate cortex (PCC), angular gyrus (ANG), aHIPPO, and pHIPPO. Schizophrenia patients also show large-scale between-network FC abnormalities of the cortico-hippocampal network, representing significantly decreased FCs between AT and PM, AT and aHIPPO, PM and aHIPPO, and aHIPPO and pHIPPO. Some aberrant FC signatures are correlated to positive, negative, total scores of PANSS and scores of cognitive items including attention/vigilance (AV), working memory (WM), verbal learning and memory (Verb_Lrng), visual learning and memory (Vis_Lrng), reasoning and problem-solving (RPS), and social cognition (SC).

Finally, Tuovinen and Hofer review 18 articles out of 158 articles from the PubMed database that matched to search criteria. Specifically, the articles published in the last 10 years (from December 2012 to November 2022) with the terms schizophrenia or psychosis, in combination with functional connectivity, resting-state fMRI (rs-fMRI), resting, resistance, resistant, nonresponsive, treatment-resistant schizophrenia or treatment-refractory schizophrenia, treatment response, response to treatment, electroconvulsive therapy, or clozapine, are considered. Their review demonstrates the frontal hypoconnectivity prior to the commencement of clozapine or riluzole treatment and shows an improvement in frontal connectivity following riluzole treatment. The authors further state that the hypoconnectivity in the fronto-temporo-occipital region may be unique to non-responders. Additionally, the observed extensive abnormal connectivity is a response to mixed treatment, and the noticeable effects on the limbic system are presumed to be induced by electroconvulsive theory.

## Conclusion

Each article focuses on a different but equally important aspect of new insights into the disorder of brain connectivity in schizophrenia. We believe this Research Topic will raise awareness within the scientific community by presenting and highlighting advances in the latest novel and emergent technologies, implementations, and applications pertaining to the brain network in schizophrenia. Finally, we would like to express our gratitude to all the authors who contributed to this Research Topic with their research. We would also like to thank the numerous experts in the field who participated in the review process and provided helpful suggestions to improve the content and presentation of the articles.

## Author contributions

WL: Writing—original draft. MA-A: Writing—original draft. AN: Writing—original draft.

